# Longitudinally Extensive Transverse Myelitis and Bilateral Pellegrini-Stieda Syndrome Coexisting in a Young Woman With Systemic Lupus Erythematosus: Report of a Rare Case

**DOI:** 10.7759/cureus.88409

**Published:** 2025-07-21

**Authors:** Mohamed Maroc, Abderrahim Lachhab, Yassine Benghali, Abdelilah Rhoul, Ahmed Amine El Oumri

**Affiliations:** 1 Faculty of Medicine, Mohammed First University, Oujda, MAR; 2 Physical Medicine and Rehabilitation, Mohammed VI University Hospital, Oujda, MAR

**Keywords:** longitudinally extensive transverse myelitis, neurogenic heterotopic ossification, pellegrini-stieda syndrome, shockwave therapy, systemic lupus erythematosus

## Abstract

Systemic lupus erythematosus is a complex autoimmune disease, with longitudinally extensive transverse myelitis representing a rare yet severe neuropsychiatric manifestation that can be its inaugural presentation. While Pellegrini-Stieda disease, characterized by ossification of the medial collateral ligament of the knee, is typically a post-traumatic condition, its occurrence in the context of neurological injury is extremely uncommon. We report a rare case of a 31-year-old woman with systemic lupus erythematosus presenting with longitudinally extensive transverse myelitis and paraplegia. This neurological manifestation was later complicated by bilateral Pellegrini-Stieda disease, an unusual musculoskeletal manifestation likely triggered by neurogenic mechanisms related to prolonged immobilization. Her symptoms improved with Extracorporeal Shockwave Therapy, a case that underscores the importance of recognizing atypical joint complications like Pellegrini-Stieda disease in immobilized Systemic lupus erythematosus patients, advocating for early multidisciplinary intervention. This rare association highlights the need for early rehabilitation strategies to prevent such complications.

## Introduction

Systemic lupus erythematosus (SLE) is a complex autoimmune disorder characterized by immune system dysregulation, leading to a broad spectrum of clinical manifestations [1]. It disproportionately affects African American women and other ethnic minority groups. The diverse neurological and psychiatric symptoms of SLE, collectively known as neuropsychiatric SLE (NPSLE), can arise from factors like vasculopathy, autoantibodies, and inflammatory mediators, underscoring SLE's extensive impact on the nervous system [1,2]. Within the broad spectrum of NPSLE, longitudinally extensive transverse myelitis (LETM) is a rare and severe form of spinal cord involvement, defined by T2-weighted magnetic resonance imaging (MRI) lesions extending over at least three contiguous vertebral segments [3]. Notably, LETM can sometimes be the inaugural presentation of SLE, preceding other systemic manifestations [4].

Pellegrini-Stieda disease (PSD) is a distinct musculoskeletal condition characterized by post-traumatic ossification at the proximal attachment of the knee's medial collateral ligament (MCL), typically diagnosed radiographically [5,6]. While commonly associated with direct or indirect knee injuries, the link between ectopic bone formation in PSD and neurological insults can be attributed to several factors. These include tissue hypoxia, hypercalcemia, altered sympathetic nerve activity, prolonged immobilization, and remobilization with frequent exercise after extended periods of immobility, mirroring the pathogenesis of neurogenic heterotopic ossification (NHO) [1,7].

To our knowledge, this combination has not been previously described in an SLE patient with LETM. This case report details a rare presentation of a young female patient with inaugural SLE-related LETM who subsequently developed bilateral PSD during her rehabilitation course. Our report highlights that PSD can manifest in neurologically compromised patients, making it a critical consideration in the differential diagnosis for new-onset knee pain, swelling, and restricted joint mobility within this specific and vulnerable population.

## Case presentation

A 31-year-old female initially presented with acute-onset paraplegia, marking the inaugural manifestation of her underlying condition. Her symptoms included concurrent saddle anesthesia, paresthesias, and sphincter dysfunction, notably a distended bladder. She also reported a history of inflammatory arthralgias, a malar rash, and oral ulcerations.

Upon examination, respiratory, cardiac, abdominal, and joint assessments were unremarkable. Neurological evaluation revealed severe motor weakness in the lower limb muscles, including the quadriceps, hamstrings, and the muscles of the lower leg and foot (Medical Research Council (MRC) Scale 1/5 strength), with preserved upper limb strength (MRC Scale 5/5). Sensory testing demonstrated reduced pain, touch, and temperature sensation below the T1 dermatome. Dermatological assessment identified erythematous, raised, and scaly skin plaques.

The patient underwent a biological workup, including a lumbar puncture. The lumbar puncture revealed clear cerebrospinal fluid (CSF) with a protein concentration of 0.29 g/L (normal range: 0.15-0.45 g/L) and no cellular reaction. The results of the blood workup are summarized in Table 1. These results indicated an inflammatory state, along with neutropenia and lymphopenia. Antinuclear antibodies (ANA) were positive with a speckled pattern.

**Table 1:** Summary of blood test results

Test	Patient Result	Normal Range	Comments
Erythrocyte Sedimentation Rate (ESR)	67 mm/1st hour	<20 mm/1st hour	Elevated, indicating inflammation
C-Reactive Protein (CRP)	25 mg/L	<5 mg/L	Elevated, indicating inflammation
Lymphocyte Count	0.8 x 10^9/L	1.0-4.0 x 10^9^/L	Lymphopenia
Neutrophil Count	1.2 x 10^9/L	2.0-7.5 x 10^9^/L	Neutropenia
Antinuclear Antibodies (ANA)	1/320 speckled pattern	-	Positive, suggestive of autoimmune disease

**Figure 1:**
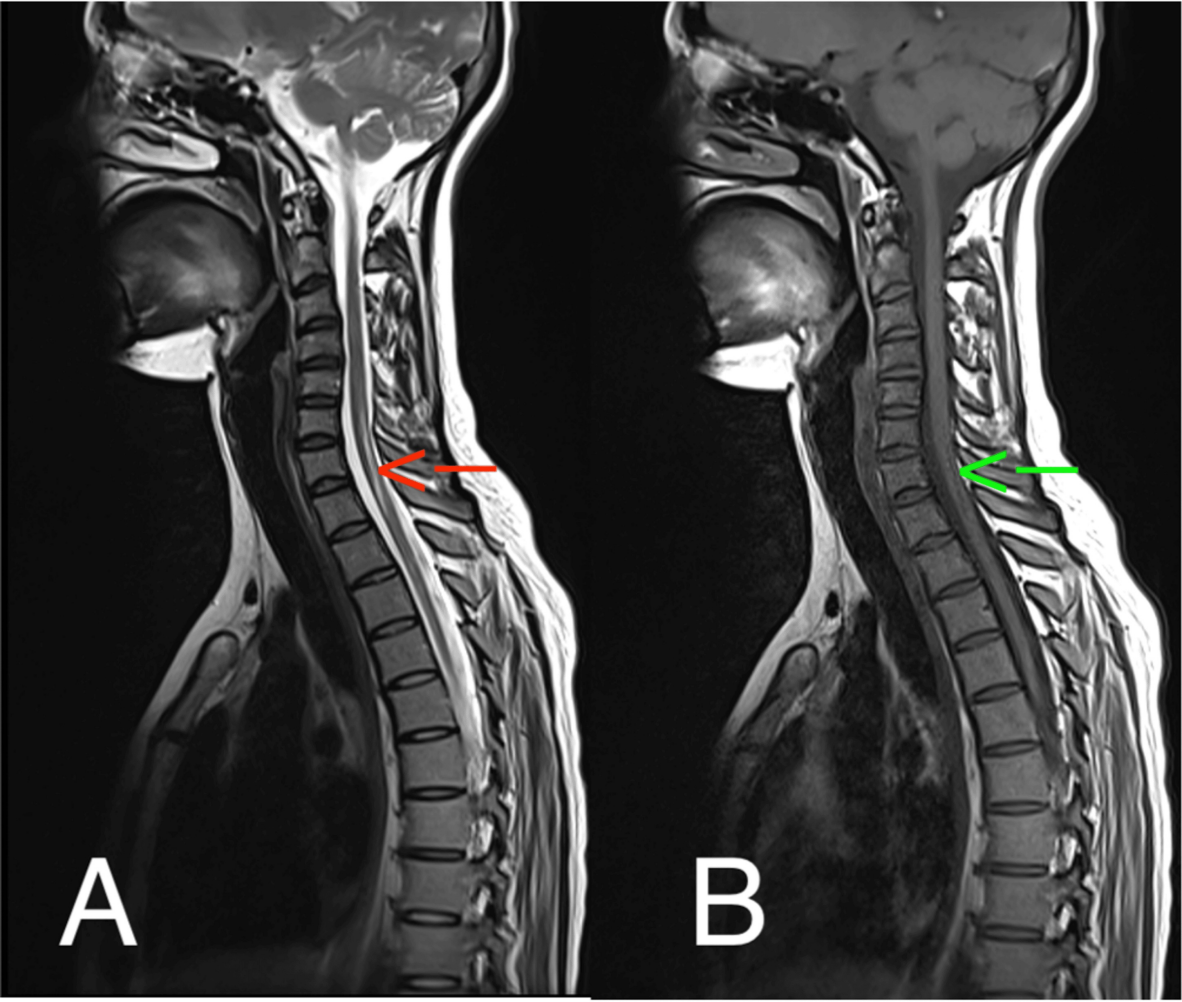
Thoracic spinal MRI demonstrating LETM (a) Sagittal T2-weighted (T2W) image shows an intramedullary hypersignal (red arrow) extending from T1 to T4 vertebral segments, consistent with edema or inflammation. (b) Corresponding sagittal T1-weighted (T1W) image displays an intramedullary hyposignal (green arrow) at the same level, reinforcing the diagnosis of myelitis. LETM: Longitudinally extensive transverse myelitis.

MRI of the spine confirmed the diagnosis of LETM, revealing a continuous lesion extending over more than three vertebral segments, consistent with its extensive nature (Figure 1).

The diagnosis of SLE, manifesting as transverse myelitis, was subsequently established based on the updated American College of Rheumatology/European League Against Rheumatism (ACR/EULAR) 2019 classification criteria. This diagnosis was supported by the presence of positive antinuclear antibodies (ANA) as an essential entry criterion, along with characteristic clinical and laboratory findings, including oral ulcerations, a malar rash, neutropenia/lymphopenia, and the neuropsychiatric manifestation of transverse myelitis, which collectively fulfilled the required scoring threshold. The transverse myelitis was attributed to SLE after careful exclusion of other etiologies.

Initial management for the LETM, in line with standard care for severe lupus myelitis, included a regimen of high-dose corticosteroids (1 mg/kg/day) and cyclophosphamide boluses, administered over a period of approximately three months. Despite these interventions and the concurrent initiation of functional rehabilitation, assessed at three months post-onset, significant neurological recovery was unfortunately not achieved, and the deficit persisted.

During her subsequent hospitalization for rehabilitation, where she engaged in day hospital sessions three times per week, comprising active and passive mobilization, stretching exercises, and assisted gait training tailored to her paraplegia, the patient developed significant pain over the medial aspects of both knees. This pain was objectively assessed using a Visual Analog Scale (VAS) of 6/10, indicating moderate discomfort, and was associated with a restricted range of motion: right knee flexion was limited to 60 degrees, and left knee flexion to 80 degrees.

**Figure 2:**
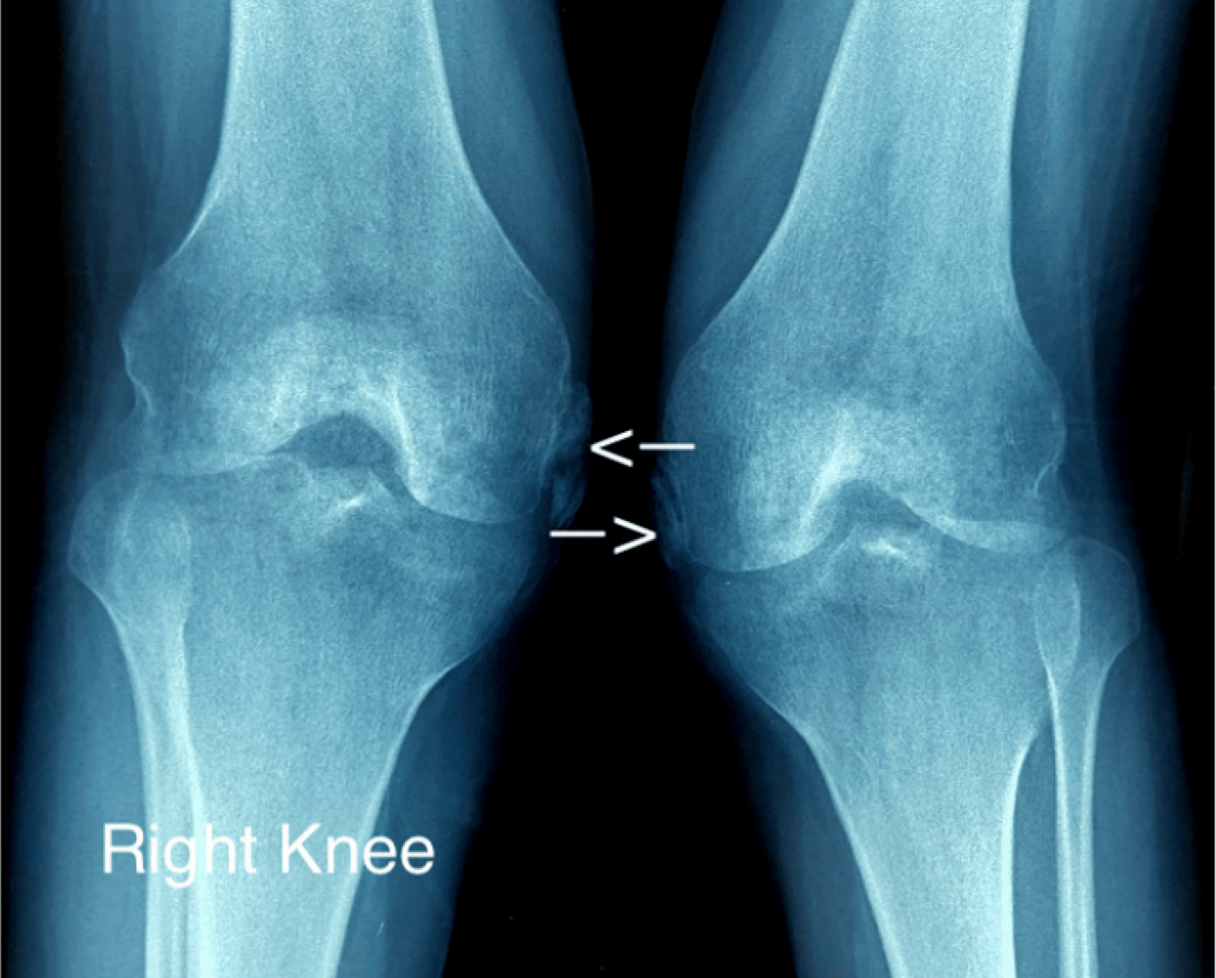
Plain radiograph of both knees showing bilateral Pellegrini-Stieda calcification This anteroposterior view of both knees strikingly demonstrates distinct bilateral heterotopic ossification (white arrows) of the MCL, adjacent to the medial femoral condyles, a notable finding in this patient.

**Figure 3:**
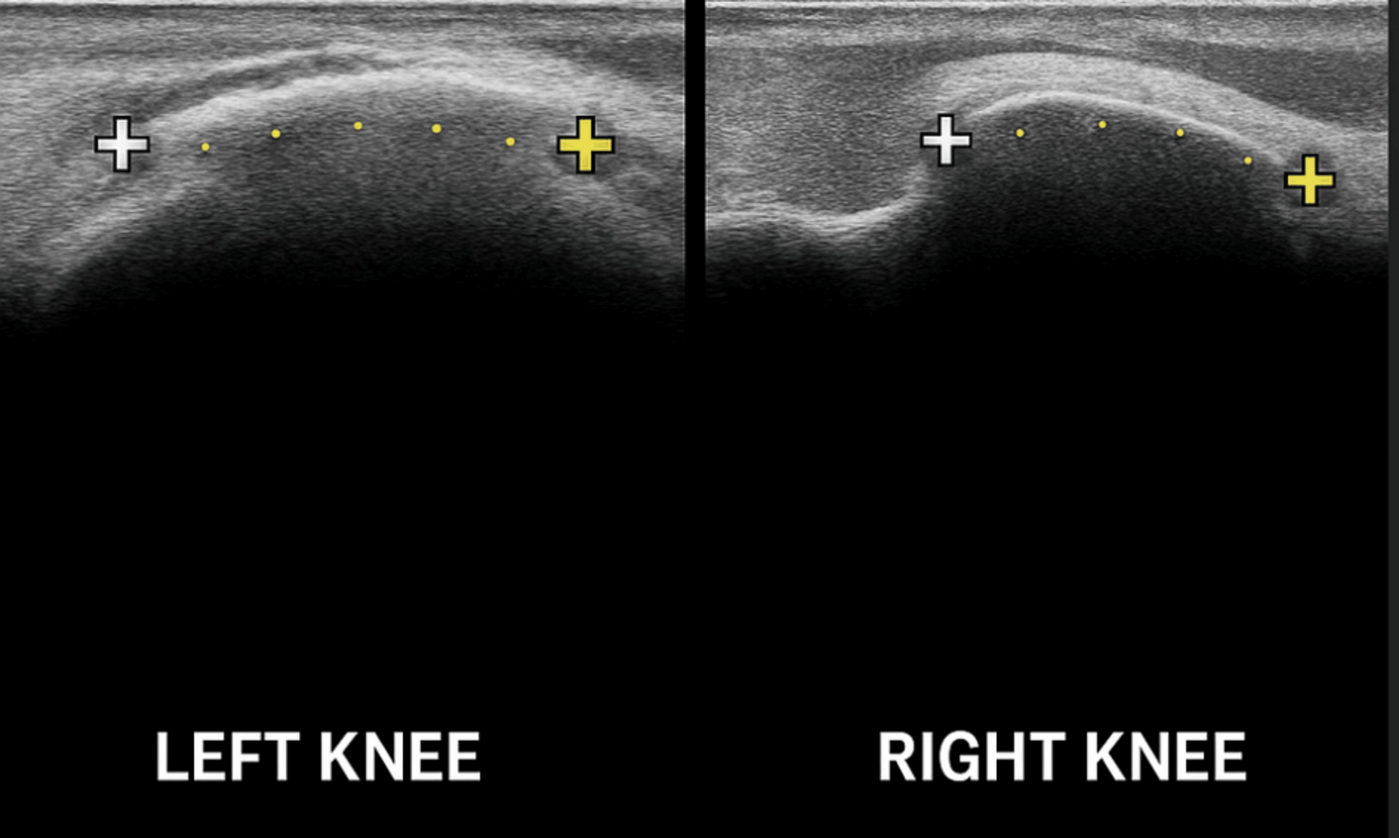
Ultrasound Image of both knees confirming Pellegrini-Stieda calcification

Given these new symptoms, both plain radiographs (Figure 2) and ultrasound examinations (Figure 3) of the knees were promptly conducted. These imaging modalities identified characteristic calcifications located at the MCL of both knees, definitively establishing the diagnosis of bilateral PSD.

Extracorporeal Shockwave Therapy (ESWT) was administered using a BTL-6000 SWT Topline machine (BTL Industries Ltd., Prague, Czech Republic). A total of twelve applications were delivered over a 12-week period to the MCL area of both knees. For each treatment session, three thousand shock waves were delivered to both knees at an intensity level of 5-6. Concurrently, the patient continued her established functional rehabilitation program. Following this intervention, the patient's pain significantly improved, with a VAS of 4/10. Her range of motion also showed favorable progress, with right knee flexion reaching 90 degrees and left knee flexion 70 degrees.

## Discussion

SLE is a complex autoimmune disorder driven by a dysfunctional immune system, leading to a wide array of clinical presentations [1]. As observed in our patient, it disproportionately affects women, particularly those of African American descent and other ethnic minority groups [1]. In 1999, the ACR introduced a nomenclature system for NPSLE, identifying 19 distinct syndromes to facilitate their recognition, diagnosis, and classification [1]. Our patient's initial presentation with acute-onset paraplegia highlights the severe and often inaugural nature of lupus myelitis, a rare but serious complication of SLE, occurring in approximately 1% to 2% of cases [2,3]. Myelitis, particularly LETM, which involves lesions spanning at least three contiguous vertebral segments on MRI, presents as a complete spinal cord syndrome characterized by bilateral sensorimotor deficits and sphincter dysfunction [3,5]. Our case further emphasizes that LETM can indeed be the initial manifestation of SLE, often preceding the full spectrum of systemic symptoms [5]. The diagnosis of SLE-related myelitis can pose challenges, necessitating a thorough exclusion of other etiologies before confirming the diagnosis based on updated classification criteria, as applied in our patient's case [2].

The subsequent development of PSD in our patient during her rehabilitation introduces a less commonly reported musculoskeletal complication in neurologically impaired individuals. PSD is precisely defined as the ossification of the MCL at or near its proximal insertion on the medial femoral condyle. Definitive diagnosis is typically achieved through radiography and confirmed by ultrasound [6,8]. Clinically, it presents with localized pain, swelling, and restricted knee mobility, consistent with our patient's symptoms [8]. The association between PSD and neurological injuries, such as spinal cord myelopathy, is rare, with limited direct reports in the literature [9].

To investigate this rarity further, a comprehensive literature review was conducted across several major databases, including Cumulative Index to Nursing and Allied Health Literature (CINAHL), PubMed, Cochrane Clinical Trials, Database of Abstracts of Reviews of Effects (DARE), Medical Literature Analysis and Retrieval System Online (MEDLINE), Scopus, Embase, and Science Direct. This focused search, spanning from January 2000 to December 2024, specifically employed the keywords: "Pellegrini-Stieda," "Traumatic Brain Injury," "Spinal Cord Injury," "Neuropathy," "Neurogenic," and "Brain Injury." This methodology yielded very limited results concerning a direct link between PSD and neurological trauma. Only three documented cases of PSD following a spinal cord injury and a single case after a traumatic brain injury were identified [5,9-11]. Despite the scarcity of direct evidence, the underlying pathophysiology of ectopic bone formation in PSD shares similarities with NHO, a process well-documented in patients with neurological insults [9,10]. Factors contributing to NHO, which may also explain the development of PSD in this context, include tissue hypoxia, hypercalcemia, altered sympathetic nerve activity, prolonged immobilization, and the initiation of remobilization with frequent exercise after extended periods of immobility [9,10]. Our case, detailing bilateral PSD in a patient with severe LETM, underscores a plausible link through these neurogenic mechanisms, highlighting a crucial consideration in patients undergoing neurological rehabilitation, even in the absence of overt trauma. In this particular case, ESWT was chosen because initial physical therapy alone had not achieved sufficient pain reduction or improvement in knee range of motion, thus warranting an escalated, non-invasive therapeutic approach to aid in both pain relief and rehabilitation progression.

Regarding management, conservative approaches for mild-to-moderate PSD include physical therapy and corticosteroid injections. For persistent symptoms, emerging therapeutic options like ultrasound-guided percutaneous calcific lavage and autologous platelet-rich plasma infiltration have been explored [8]. Notably, ESWT, as utilized effectively in our patient, is recognized as a beneficial non-invasive treatment for MCL ossifications, demonstrating favorable outcomes in pain reduction and improved range of motion [11]. When conservative measures fail, surgical excision of the ossified fragment, often combined with MCL repair, remains a recommended option [8].

This case report underscores the importance of vigilance in recognizing rare musculoskeletal complications like PSD in patients undergoing neurological rehabilitation, especially when a clear traumatic etiology is absent. The occurrence of bilateral PSD in a patient with inaugural SLE-related LETM highlights the complex interplay between autoimmune disease, neurological deficit, and ectopic ossification, necessitating comprehensive assessment and tailored multidisciplinary management to optimize patient outcomes.

## Conclusions

This case report details the rare and complex presentation of a young female patient whose SLE initially manifested as LETM. This unique trajectory underscores that lupus myelitis, particularly its severe and extensive form LETM, must be considered a potential inaugural symptom of SLE, even in the absence of other established diagnostic criteria. The subsequent development of bilateral PSD during her rehabilitation, while uncommon, highlights a plausible link to NHO mechanisms, influenced by factors such as prolonged immobilization and subsequent mobilization. This association necessitates heightened clinical suspicion for new-onset joint pain and mobility limitations in patients undergoing neurological rehabilitation, even without a clear traumatic event. Our report further reinforces the effectiveness of ESWT as a valuable non-invasive treatment modality for managing the pain and improving the range of motion associated with PSD, ultimately emphasizing that comprehensive assessment and a multidisciplinary approach are paramount for optimizing outcomes in such intricate cases, ensuring rare musculoskeletal complications are not overlooked in patients with complex autoimmune and neurological conditions.
